# The Associations of Lymphocyte Ratio and Neutrophil Ratio on Liver Dysfunction in COVID-19 Patients

**DOI:** 10.3389/fimmu.2021.717461

**Published:** 2021-09-06

**Authors:** Fang Liu, Hong Liu, Wen-Yan Yu, Zhan Liu, Xia Zhang, Yi Wang, Liang-Bin Miao, Zhao-Yi Li, Jin-Song Huang, Jian-Feng Bao

**Affiliations:** ^1^Institute of Hepatology and Epidemiology, Affiliated Hangzhou Xixi Hospital, Zhejiang University School of Medicine, Hangzhou, China; ^2^Department of Pathology, Affiliated Hangzhou Xixi Hospital, Zhejiang University School of Medicine, Hangzhou, China; ^3^Medical Laboratory, Affiliated Hangzhou Xixi Hospital, Zhejiang University School of Medicine, Hangzhou, China; ^4^Department of Anesthesiology, Affiliated Hangzhou Xixi Hospital, Zhejiang University School of Medicine, Hangzhou, China; ^5^Department of Hepatology, Affiliated Hangzhou Xixi Hospital, Zhejiang University School of Medicine, Hangzhou, China

**Keywords:** COVID-19, inflammation, liver dysfunction, lymphocyte, neutrophil

## Abstract

Data on the impact of lymphocytes and neutrophils on the incidence of liver dysfunction in COVID-19 patients are limited. This study aimed to investigate the lateral and longitudinal associations of lymphocyte ratio (LR) and neutrophil ratio (NR) on liver dysfunction in COVID-19 patients. We tested 1,409 blood samples from 245 COVID-19 patients in China between January 2020 and June 2021. The lateral U-shaped relationships, determined by smooth curve fitting and the piecewise-linear mixed-effect model, were observed between LR, NR, and AST and the incidence of AST-linked liver dysfunction, with the threshold cutoffs of 26.1 and 62.0, respectively. Over the 1,409 tests, the LR ≤ 26.1 and NR ≥ 62.0 related to the occurrence of mild liver dysfunction (HR: 1.36; 95% CI: 1.01, 1.82), moderate liver dysfunction (HR: 1.37; 95% CI: 1.01, 1.85), and severe liver dysfunction (HR: 1.72; 95% CI: 1.02, 2.90). For the patients with preexisting AST ≥ 35 U/L, the baseline LR ≤ 26.1 and NR ≥ 62.0 (b.LLCHN) groups had a fully adjusted 8.85-, 7.88-, and 5.97-fold increased risk of mild and moderate liver dysfunction after being hospitalized of 3, 6, and 9 days compared to the baseline LR > 26.1 and NR < 62.0 (b.normal) groups. Severe liver dysfunction only presents significant differences after being adjusted for age, sex, and BMI. Consistently, Kaplan–Meier analyses showed that b.LLCHN reflects a better predictive value for different subsequent magnitude liver dysfunctions after admission of 3 and 6 days. To improve liver function in patients with preexisting AST ≥35 U/L, future management strategies should pay more attention to baseline LR ≤ 26.1 and NR ≥ 62.0 patients.

## Introduction

Coronavirus disease 2019 (COVID-19)-related liver damage has significant associations with patient severity and mortality (1–4). Liver dysfunction events are the most notable additional feature observed in COVID-19 patients (5), and the prevalence rate of liver injury is reported in the range of 21.5%–45.7% in these individuals (6). Therefore, detecting new readily available risk factors to inform the early prevention of liver damage becomes the current clinical practice of urgent needs.

Immune-mediated inflammation plays an important role in the initiation and progression of liver damage in COVID-19 patients (7). Lymphocytes and neutrophils can infiltrate the injured liver *via* the circulatory system, stimulating further inflammation cytokine production and promoting tissue damage (8). Lymphopenia and high neutrophil count associated with severe disease in COVID-19 patients have been reported in previous studies (9–11). However, limited data are available on the impact of blood lymphocyte and neutrophil concentrations on the incidence of liver injury. By far, only one study carried out by Lei et al. synchronously assessed the correlations of lymphocytes and neutrophils with liver function (4). In this report, a high risk of elevated aspartate aminotransferase (AST), which appears to reflect actual hepatic injury (12), is associated with the normal lower limit of lymphocytes <1.1 × 10^9^/l (OR: 2.21; 95% CI: 1.89, 2.58) and the normal upper limit of neutrophils >6.3 × 10^9^/l (OR: 1.60; 95% CI: 1.31, 1.95) (4). However, the lateral dynamic associations of lymphocytes and neutrophils with AST have not been estimated sufficiently in this report. Moreover, there is little research on at what cutoffs of lymphocytes and neutrophils abnormalities of liver function occur and how those may relate to abnormalities.

Here, the present study enrolled 245 patients with COVID-19 in China to address the following two questions:

1. What are the dynamic relations of liver dysfunction with lymphocytes and neutrophils in COVID-19 patients exposed to lopinavir (LPV), arbidol hydrochloride (ABI), interferon (IFN), or ribavirin (RBV) during a lateral observation period?

The drugs mentioned above are the commonly used hepatotoxic antiviral drugs in China (13, 14). We were particularly interested in assessing the saturation and threshold effects of lymphocytes and neutrophils on AST, which mirrors disease severity and mortality and appears to reflect true hepatic injury (12). The variable screening model was used to confirm the liver dysfunction-related covariables (15).

2. What are the longitudinal associations of baseline lymphocytes and neutrophils with subsequent liver dysfunction?

We hypothesized that low lymphocytes and high neutrophils at baseline could jointly and independently increase the risk of liver dysfunction.

## Materials and Methods

### Study Participants

The present study is a combination design of lateral observation and longitudinal analysis. We tested 1,409 blood samples from 245 COVID-19 patients at the infectious diseases department at Affiliated Hangzhou Xixi Hospital, Zhejiang University School of Medicine (Zhejiang, Southeast China) from January 2020 to June 2021. The patients diagnosed with COVID-19 were screened for eligibility. This study was approved by the Institutional Review Board of Xixi Hospital. Informed consent was not required due to the retrospective nature of this study.

### Inclusion Criteria and Exclusion Criteria

The inclusion criterion was patients confirmed as COVID-19 by a positive real-time reverse transcriptase-polymerase chain reaction (RT-PCR) test of SARS-CoV-2 RNA, which was conducted according to the methods described previously (16). The exclusion criterion was COVID-19 patients without exposure of any following antiviral drugs: LPV, ABI, IFN, or RBV.

### Definitions

Three liver outcomes were defined: mild liver dysfunction, moderate liver dysfunction, and severe liver dysfunction. Mild liver dysfunction was defined as AST ≥35 U/L. To further describe the liver function, we defined moderate liver dysfunction as AST ≥35 U/L combined with any parameter being greater than the upper limits of the normal (ULN) values of alanine transaminase (ALT ≥40 U/L), γ-glutamyl transpeptidase (GGT ≥45 U/L), and total bilirubin (TBIL ≥20.52 µmol/L). The levels of AST ≥35 U/L combined with ALT ≥3×ULN and/or GGT, TBIL ≥2×ULN, were defined as severe liver dysfunction (2).

### Covariate Selection and Study Design

All the data were collected and reviewed by an independent Medical Big Data Processing Team in our hospital. Considering the lack of key covariables that can lead to unreliable results, a total of 22 variables were confirmed as covariate by their correlations with AST and AST-linked liver dysfunction (*p* < 0.01) or a change in regression coefficients of more than 10% (**Table S1**) (15).

#### Lateral Observation Study Design

Eleven covariates [age, sex, body mass index (BMI), SBP, highest temperature, smoking status, alcohol, chest congestion, liver cirrhosis, HBV, and clinical classification] of admission were selected as fixed variables. Eleven serum indexes [white blood cell count (WBC), rapid C-reactive protein (RCP), serum amyloid A protein (SAA), alkaline phosphatase (ALP), activated partial thromboplastin time (APTT), glomerular filtration rate (GFR), lipoprotein A (LppA), hemoglobin (Hb), sodium concentration, actual base excess (ABE), and anion gap (AG)] were chosen as the time-varied variables.

During the period, a total of 1,409 measurements were conducted in 245 COVID-19 patients; the levels of AST, ALT, GGT, TBIL, lymphocyte ratio (LR), neutrophil ratio (NR), and the above 11 serum indexes were tested simultaneously according to each patient’s condition. All the measurements were divided into two categories according to the values of LR and NR: LLCHN group (low LR combined with high NR, LR ≤ 26.1, and NR ≥ 62.0) and normal group (LR > 26.1 and NR < 62.0). Triage criteria followed those confirmed by the cutoffs of LR and NR from piecewise-linear multiple regression analysis.

#### Longitudinal Study Design

To detect the prospective relations of LR and NR with subsequent liver dysfunction, a longitudinal study design was conducted as follows:

1. The peak values of AST, LR, NR, and the above 11 serum indexes within 3 days of admission to the hospital were picked out and defined as baseline variables.

2. Liver endpoints included mild liver dysfunction, moderate liver dysfunction, and severe liver dysfunction that occurred after 3, 6, and 9 days since admission, which were named 3-, 6-, and 9-day liver dysfunction, respectively.

### Statistical Analysis

#### Lateral Observation Analysis

A multivariable smooth curve fitting function was used to detect the non-linear association of LR and NR with AST. Piecewise-linear multiple regression analysis based on the generalized additive mixed model was further utilized to examine the threshold and saturation effects of LR and NR on AST, and the results were presented as regression coefficients [exp(*β*)] with their 95% CI (**Table S2**). In this analysis, the threshold level was determined by choosing the turning point of the smooth curve, which utilized a maximum likelihood model through a recursion method; a log-likelihood ratio test was performed simultaneously to examine the statistical significance (17).

#### Longitudinal Analysis

The longitudinal associations of baseline LR and NR with liver dysfunction were estimated in Cox models. Patients were stratified into two groups: baseline LR > 26.1 and NR < 62.0 (b.normal group) and baseline LR ≤ 26.1 and NR ≥ 62.0 (low lymphocyte ratio combined with the high neutrophil ratio at baseline, b.LLCHN group).

Descriptive analysis was used to compare the differences in demographics, clinical manifestations, laboratory factors, and antiviral usage between the b.normal group and the b.LLCHN group (**Tables 1** and **S3**). Categorical variables were described as frequency (percent, %), and continuous variables were presented as median (interquartile range, IQR). Variables were compared using the chi-squared test, one-way ANOVA, or Student’s t-test as appropriate. Data on covariates were not available; a missing value category was used in the analysis to reduce statistic bias (18). Kaplan–Meier analysis was used to compare the cumulative incidence of liver dysfunction endpoints between the b.normal group and the b.LLCHN group.

**Table 1:** Characteristics of 245 COVID-19 patients within 3 days of admission.

Characteristics	b.Normal group	b.LLCHN group	*p* value
Number (%)	146 (59.6%)	99 (40.4%)	
Age	33.0 (26.0–43.8)	39.0 (32.5–51.0)	<0.001
Female	49 (33.6%)	46 (46.5%)	0.042
BMI	22.9 (20.5–25.7)	22.4 (20.1–24.8)	0.258
SBP	129.0 (117.0–138.0)	130.0 (116.0–140.0)	0.354
Smoking	29 (20.0%)	9 (9.1%)	0.021
Alcohol	43 (29.5%)	22 (22.2%)	0.208
Highest temperature	37.0 (36.5–37.5)	37.0 (36.8–37.8)	0.075
Chest congestion	3 (2.1%)	8 (8.1%)	0.026
Liver cirrhosis	1 (0.7%)	0 (0.0%)	0.409
HBV	8 (5.5%)	4 (4.0%)	0.600
**Clinical classification**			0.001
Asymptomatic	54 (37.0%)	24 (24.2%)	
Mild	26 (17.8%)	10 (10.1%)	
Moderate	65 (44.5%)	57 (57.6%)	
Severe	1 (0.7%)	8 (8.1%)	
**Inflammatory indicators**			
WBC (×10^9^/L)	5.9 (4.8–7.1)	7.0 (5.8–8.5)	<0.001
RCP (mg/L)	5.0 (1.0–10.0)	6.0 (2.0–28.5)	0.082
SAA (mg/L)	18.0 (5.0–88.0)	51.0 (11.5–137.0)	0.006
Lymphocyte ratio (%)	34.5 (29.5–39.9)	20.3 (16.8–22.8)	<0.001
Neutrophil ratio (%)	57.7 (51.6–63.3)	73.0 (67.7–78.5)	<0.001
**Liver biochemical indicators**			
ALT (U/L)	20.0 (13.0–32.0)	19.0 (12.5–39.0)	0.882
AST (U/L)	22.0 (18.0–27.0)	24.0 (17.0–29.5)	0.566
GGT (U/L)	23.0 (16.0–35.0)	22.0 (15.5–36.5)	0.846
TBIL (µmol/L)	12.8 (9.5–18.3)	14.5 (9.6–19.8)	0.393
ALP (U/L)	77.0 (62.0–90.0)	75.0 (63.0–89.5)	0.799
APTT (s)	28.0 (26.6–30.0)	27.9 (26.6–31.4)	0.488
**Others**			
GFR (mL/min)	116.0 (103.0–127.0)	112.0 (100.2–128.5)	0.397
LppA (mg/L)	110.8 (64.2–324.9)	155.2 (76.8–298.2)	0.200
Hb (g/L)	148.0 (134.0–156.0)	140.0 (130.0–152.0)	0.015
Sodium concentration (mmol/L)	138.7 (137.6–140.1)	138.4 (136.3–139.8)	0.102
ABE (mmol/L)	0.7 (-0.5–1.7)	0.4 (-0.7–1.8)	0.785
AG (mmol/L)	10.0 (9.0–13.1)	10.7 (8.9–12.9)	0.561

Data are n (%) or median (IQR) unless otherwise indicated. Participants with the peak values of lymphocyte ratio ≤26.1 and neutrophil ratio ≥62.0 within 3 days of admission were classified as b.LLCHN group, and others were classified as b.Normal group.

BMI, body mass index; HBV, hepatitis B Virus; WBC, white blood cell count; RCP, rapid C-reactive protein; SAA, serum amyloid A protein; ALT, alanine aminotransferase; AST, aspartate aminotransferase; GGT, γ-glutamyl transferase; TBIL, total bilirubin; ALP, alkaline phosphatase; APTT, activated partial thromboplastin time; GFR, glomerular filtration rate; LppA, lipoprotein A; Hb, hemoglobin; ABE, actual base excess; AG, anion gap; b.LLCHN, low lymphocyte ratio complicated with high neutrophil ratio at baseline.

A result was considered statistically significant when the two-tailed *p* value was below 0.05. R software version 3.6.3 (www.R-project.org) was used for all statistical analyses.

## Results

### Subject Characteristics and Covariate Selection

**Figure 1:**
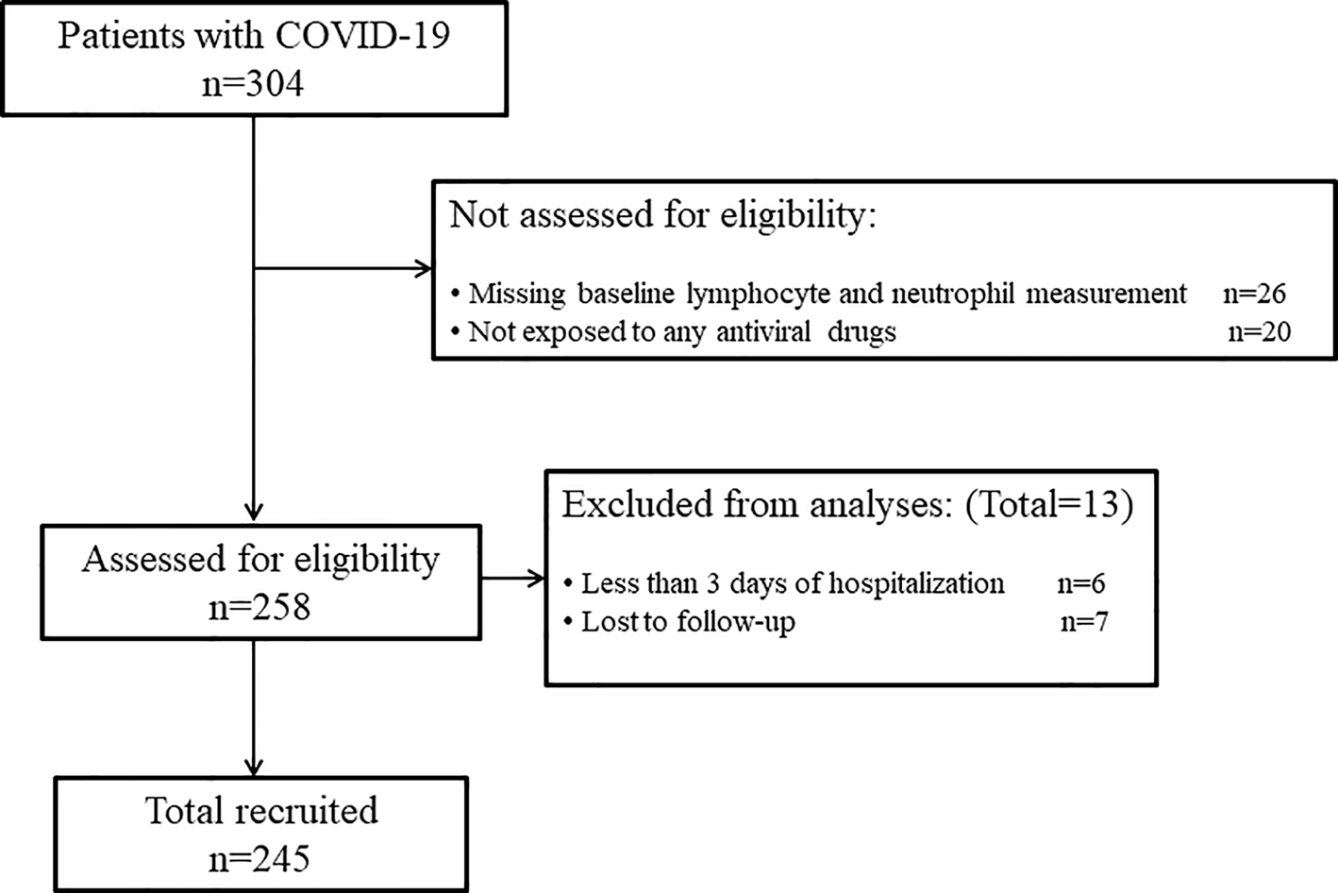
Flowchart of the included COVID-19 patients.

A total of 304 patients with confirmed COVID-19 were enrolled in this research. After reviewing the medical records, we excluded 59 patients based on the following criteria: missing baseline LR and NR (n = 26), no exposure to any antiviral drugs (n = 20), less than 3 days of hospitalization (n = 6), and lost to follow-up (n = 7). Thus, 245 patients were eventually included in this study (**Figure 1**). Of these patients, 107 (43.7%) experienced mild liver dysfunction, 99 (40.4%) experienced moderate liver dysfunction, and 50 (20.4%) experienced severe liver dysfunction over a median follow-up of 1.9 (IQR, 0.9–4.0) weeks (data not shown).

The demographics, clinical features, peak values of laboratory factors within 3 days of admission, and antiviral drug usage between the b.normal group and the b.LLCHN group are shown in **Tables 1** and **S3**, respectively. The characteristics of fixed factors on admission and time-varied laboratory indicators during the lateral observation period are shown in **Table S4**.

**Figure 2:**
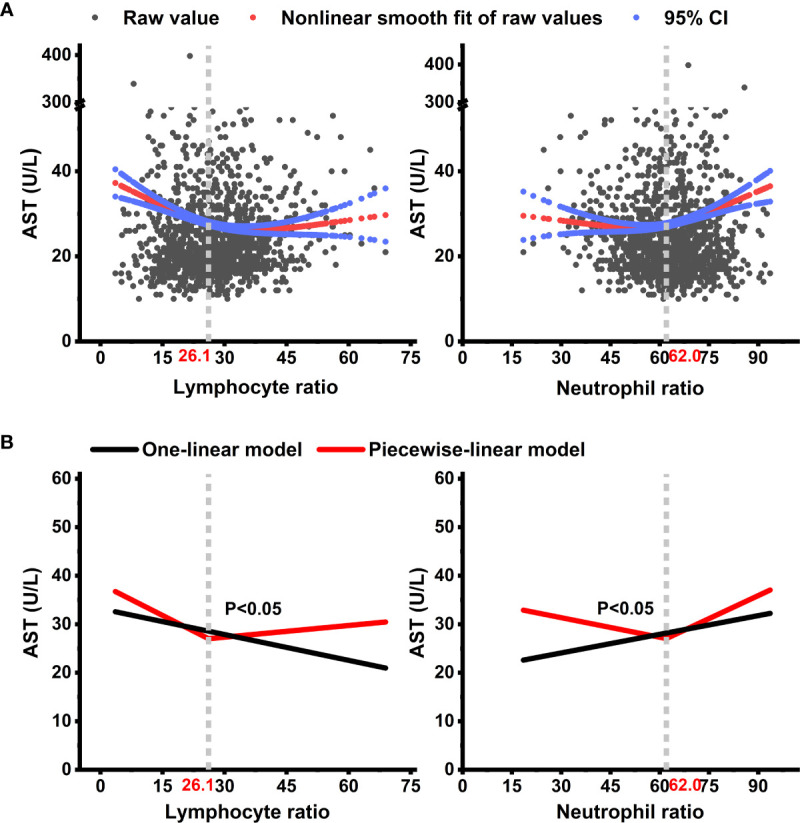
Non-linear trajectories of AST with the changes of lymphocyte ratio and neutrophil ratio during the lateral observation period among COVID-19 patients. Non-linear trajectories of AST with the changes of lymphocyte ratio and neutrophil ratio can be approximated with a piecewise-linear mixed effect model. **(A)** shows the adjusted smooth fit of AST data. **(B)** shows the fit from the adjusted one-linear and adjusted piecewise-linear mixed-effect models. Models adjusted for fixed covariates including age, sex, body mass index (BMI), SBP, smoking, alcohol, highest temperature, chest congestion, liver cirrhosis, HBV, and clinical classification on admission, and time-varied covariates including white blood cell count (WBC), rapid C-reactive protein (RCP), serum amyloid A protein (SAA), alkaline phosphatase (ALP), activated partial thromboplastin time (APTT), glomerular filtration rate (GFR), lipoprotein A (LppA), hemoglobin (Hb), sodium concentration, actual base excess (ABE), and anion gap (AG) during the lateral observation period. AST, aspartate transaminase; CI, confidence interval.

As shown in **Table 1** and **Figure 2**, enrolled patients were divided into b.normal group and b.LLCHN group according to the cutoff values of LR ≤ 26.1 and NR ≥ 62.0 from the piecewise-linear multiple regression analyses. Of these individuals, 99 (40.4%) were assigned to the b.LLCHN group. Participants in this group were older (median age: 39 years vs. 33 years, respectively; *p* < 0.001), had a higher proportion of females (46.5% vs. 33.6%, respectively; *p* = 0.042), chest congestion (8.1% vs. 2.0%, respectively; *p* = 0.026), and severe patients (8.1% vs. 0.7%, respectively; *p* < 0.001), and had a lower smoking (9.1% vs. 20.0%, respectively; *p* = 0.021) compared to the b.normal group. There were significant differences in most inflammatory indicators in the two groups. Patients in the b.LLCHN group had higher levels of WBC (median value: 7.0 vs. 5.9, respectively; *p* < 0.001), SAA (median value: 51.0 vs. 18.0, respectively; *p* = 0.006), NR (median value: 73.0 vs. 57.7, respectively; *p* < 0.001), and lower LR concentration (median value: 20.3 vs. 34.5, respectively; *p* < 0.001) compared to the b.normal group. A lower Hb (median value: 140.0 vs. 148.0, respectively; *p* = 0.015) was also observed in patients of the b.LLCHN group compared to the b.normal group. There were no significant differences in liver function, renal function, and antiviral usage between the b.normal group and the b.LLCHN group.

**Table S1** depicts the associations of each covariate with the outcomes of interest. A total of 22 variables were selected as covariates following the criterion that each variate significantly related with at least one liver outcome of interest (p < 0.01) or a change in regression coefficients of more than 10%. The lateral observation showed that only SAA is above the normal upper limit during the entire period (**Table S4**).

### Non-Linear Association of Lymphocyte and Neutrophil Ratio With AST and AST-Linked Liver Dysfunction

The non-linear associations of AST with LR and NR are presented in **Figure 2A**. U-shaped association between LR and AST was observed in the adjusted smooth curve and was also observed between NR and AST. As shown in **Figure 2B**, the turning points of LR and NR were 26.1 and 62.0, respectively. **Table S2** shows the threshold effects of LR and NR on AST. The adjusted exp(*β*) was −0.53 (95% CI: −0.84, −0.22; *p* < 0.05) for LR ≤ 26.1 and 0.34 (95% CI: 0.12, 0.57; *p* < 0.05) for NR ≥ 62.0 in the piecewise-linear regression model. The AST changed significantly with increased LR (−0.17, *p* < 0.05) in the one-linear model. The log-likelihood ratio test between the two models suggested that the non-linear fitted trajectory of AST was better than a single linear fit across the entire period (**Figure 2B** and **Table S2**, *p* < 0.05 for all).

### Lateral Observation Analysis in the COX Model

In this study, a total of 1,409 measurements for AST were conducted from January 2020 to June 2021. From the lateral observation analyses, LLCHN was significantly associated with liver dysfunction events. The incidence of mild liver dysfunction, moderate liver dysfunction, and severe liver dysfunction significantly increased to 1.36 (95% CI: 1.01, 1.82), 1.37 (95% CI: 1.01, 1.85), and 1.72 (95% CI: 1.02, 2.90) in the LLCHN group compared to the normal group after the adjustment of 22 covariates (**Table 2**). In the LLCHN group, for a per-SD increment in LR, the risk of mild liver dysfunction, moderate liver dysfunction, and severe liver dysfunction decreased to 0.58 (95% CI: 0.39, 0.85), 0.56 (95% CI: 0.38, 0.83), and 0.37 (95% CI: 0.18, 0.77), respectively, and for a per-SD increment in NR, the incidence of the mild and moderate liver dysfunction increased to 1.57 (95% CI: 1.08, 2.27) and 1.55 (95% CI: 1.05, 2.29), respectively, in the adjusted I model (**Table 3**).

**Table 2:** Lateral relations of LLCNH to the incidence of liver dysfunction events in the COX model.

	Unadjusted	Adjusted I	Adjusted II
	HR (95% CI)	HR (95% CI)	HR (95% CI)
**Mild liver dysfunction**	No. M = 1409	No. M = 1409	No. M = 1409
	1.44 (1.12, 1.84)^*^	1.56 (1.20, 2.03)^*^	1.36 (1.01, 1.82)^*^
**Moderate liver dysfunction**	No. M = 1382	No. M = 1382	No. M = 1382
	1.50 (1.16, 1.96)^*^	1.63 (1.24, 2.16)^*^	1.37 (1.01, 1.85)^*^
**Severe liver dysfunction**	No. M = 1239	No. M = 1239	No. M = 1239
	1.37 (0.89, 2.12)	1.60 (1.01, 2.54)^*^	1.72 (1.02, 2.90)^*^

Unadjusted: unadjusted for any covariables.

Adjusted I: adjusted for age, sex, and body mass index (BMI) on admission.

Adjusted II: adjusted for fixed covariates including age, sex, body mass index (BMI), SBP, smoking, alcohol, highest temperature, chest congestion, liver cirrhosis, HBV, and clinical classification on admission, and time-varied covariates including white blood cell count (WBC), rapid C-reactive protein (RCP), serum amyloid A protein (SAA), alkaline phosphatase (ALP), activated partial thromboplastin time (APTT), glomerular filtration rate (GFR), lipoprotein A (LppA), hemoglobin (Hb), sodium concentration, actual base excess (ABE), and anion gap (AG) during the lateral observation period.

LLCNH, low lymphocyte ratio complicated with high neutrophil ratio (lymphocyte ratio ≤26.1 and neutrophil ratio ≥62.0); HR, hazard ratio; CI, confidence interval; No. M, the number of measurements.

^*^The incidence of liver dysfunction in the LLCHN group was significantly different compared to the normal group.

**Table 3:** Lateral associations of the incidence of liver dysfunction events with per-SD increment in lymphocyte ratio or neutrophil ratio in the LLCHN group.

	Unadjusted	Adjusted I	Adjusted II
	HR (95% CI)	HR (95% CI)	HR (95% CI)
**Mild liver dysfunction**	No. M = 612	No. M = 612	No. M = 612
LR (per-SD increment)	0.55 (0.39, 0.77)^*^	0.51 (0.35, 0.73)^*^	0.58 (0.39, 0.85)^*^
NR (per-SD increment)	1.44 (1.00, 2.06)^*^	1.57 (1.08, 2.27)^*^	1.26 (0.84, 1.88)
**Moderate liver dysfunction**	No. M = 603	No. M = 603	No. M = 603
LR (per-SD increment)	0.53 (0.37, 0.77)^*^	0.49 (0.34, 0.71)^*^	0.56 (0.38, 0.83)^*^
NR (per-SD increment)	1.42 (0.97, 2.07)	1.55 (1.05, 2.29)^*^	1.32 (0.87, 2.01)
**Severe liver dysfunction**	No. M = 534	No. M = 534	No. M = 534
LR (per-SD increment)	0.50 (0.27, 0.92)^*^	0.44 (0.23, 0.84)^*^	0.37 (0.18, 0.77)^*^
NR (per-SD increment)	1.66 (0.85, 3.24)	1.74 (0.85, 3.54)	1.99 (0.90, 4.41)

Unadjusted: unadjusted for any covariables.

Adjusted I: adjusted for age, sex, and body mass index (BMI) on admission.

Adjusted II: adjusted for fixed covariates including age, sex, body mass index (BMI), SBP, smoking, alcohol, highest temperature, chest congestion, liver cirrhosis, HBV, and clinical classification on admission, and time-varied covariates including white blood cell count (WBC), rapid C-reactive protein (RCP), serum amyloid A protein (SAA), alkaline phosphatase (ALP), activated partial thromboplastin time (APTT), glomerular filtration rate (GFR), lipoprotein A (LppA), hemoglobin (Hb), sodium concentration, actual base excess (ABE), and anion gap (AG) during the lateral observation period.

LLCNH, low lymphocyte ratio complicated with high neutrophil ratio (lymphocyte ratio ≤26.1 and neutrophil ratio ≥62.0); HR, hazard ratio; CI, confidence interval; No. M, the number of measurements.

^*^A result that was considered statistically significant.

### Longitudinal Analysis in the COX Model

**Table 4** shows threshold effects of baseline LR ≤ 26.1 and NR ≥ 62.0 on the risk of subsequent liver dysfunction after 3, 6, and 9 days since admission from the univariate and multivariate analyses. Compared to the b.normal group, patients with baseline AST ≥ 35 U/L had a significantly higher risk of liver dysfunction in the b.LLCHN group. The risk of 3-, 6-, and 9-day mild liver dysfunction significantly increased to 8.85 (95% CI: 2.70, 28.99), 7.88 (95% CI: 2.30, 27.03), and 5.97 (95% CI: 1.51, 23.67) in the b.LLCHN group in the adjusted II model. Similar results were observed for moderate liver dysfunction. The risks of severe liver dysfunction were at least 3.5-fold greater than the b.normal group, after all three time-points in the b.LLCHN group in adjusted I models. The risks of severe liver dysfunction in the adjusted II model were also increased but not significant. Notably, there were no significant differences for all liver outcomes of interest between the b.normal group and the b.LLCHN group in patients with baseline AST <35 U/L.

**Table 4:** Longitudinal threshold effect analyses for the relations between b.LLCHN and the incidence of 3-, 6-, and 9-day liver dysfunction.

	3 days after admission		6 days after admission		9 days after admission	
	AST <35 U/L	AST ≥35 U/L	AST <35 U/L	AST ≥35 U/L	AST <35 U/L	AST ≥35 U/L
	HR (95% CI)	HR (95% CI)	HR (95% CI)	HR (95% CI)	HR (95% CI)	HR (95% CI)
**Mild liver dysfunction**						
Unadjusted	1.12 (0.78, 1.59)	2.13 (1.27, 3.58)^*^	1.08 (0.75, 1.53)	1.85 (1.09, 3.15)^*^	1.13 (0.77, 1.64)	1.48 (0.85, 2.59)
Adjusted I	1.24 (0.86, 1.80)	3.14 (1.73, 5.69)^*^	1.19 (0.82, 1.73)	2.75 (1.48, 5.09)^*^	1.32 (0.88, 1.97)	2.44 (1.25, 4.75)^*^
Adjusted II	1.11 (0.71, 1.73)	8.85 (2.70, 28.99)^*^	1.14 (0.69, 1.86)	7.88 (2.30, 27.03)^*^	1.22 (0.75, 1.99)	5.97 (1.51, 23.67)^*^
**Moderate liver dysfunction**						
Unadjusted	1.13 (0.78, 1.64)	2.13 (1.27, 3.58)^*^	1.11 (0.76, 1.61)	1.85 (1.09, 3.15)^*^	1.14 (0.77, 1.68)	1.48 (0.85, 2.59)
Adjusted I	1.33 (0.90, 1.98)	3.14 (1.73, 5.69)^*^	1.31 (0.88, 1.94)	2.75 (1.48, 5.09)^*^	1.37 (0.90, 2.07)	2.44 (1.25, 4.75)^*^
Adjusted II	1.21 (0.75, 1.95)	8.85 (2.70, 28.99)^*^	1.34 (0.78, 2.29)	7.88 (2.30, 27.03)^*^	1.26 (0.76, 2.09)	5.97 (1.51, 23.67)^*^
**Severe liver dysfunction**						
Unadjusted	0.90 (0.46, 1.79)	2.86 (1.32, 6.17)^*^	0.90 (0.46, 1.79)	2.67 (1.23, 5.81)^*^	0.99 (0.49, 1.98)	2.06 (0.90, 4.74)
Adjusted I	1.06 (0.52, 2.17)	3.83 (1.63, 9.02)^*^	1.06 (0.52, 2.17)	3.50 (1.46, 8.40)^*^	1.16 (0.56, 2.41)	3.53 (1.34, 9.29)^*^
Adjusted II	1.30 (0.47, 3.63)	6.73 (0.96, 47.34)	1.78 (0.57, 5.50)	6.45 (0.89, 46.95)	1.30 (0.45, 3.73)	3.74 (0.23, 60.25)

Unadjusted: unadjusted for any covariables.

Adjusted I: adjusted for age, sex, and body mass index (BMI) at baseline.

Adjusted II: adjusted for age, sex, body mass index (BMI), SBP, smoking, alcohol, highest temperature, chest congestion, liver cirrhosis, HBV, clinical classification, white blood cell count (WBC), rapid C-reactive protein (RCP), serum amyloid A protein (SAA), alkaline phosphatase (ALP), activated partial thromboplastin time (APTT), glomerular filtration rate (GFR), lipoprotein A (LppA), hemoglobin (Hb), sodium concentration, actual base excess (ABE), and anion gap (AG) at baseline.

b.LLCNH, low lymphocyte ratio complicated with high neutrophil ratio at baseline (baseline lymphocyte ratio ≤26.1 and neutrophil ratio ≥62.0); HR, hazard ratio; CI, confidence interval.

^*^The incidence of liver dysfunction in the b.LLCHN group was significantly different compared to the b.normal group.

**Figure 3:**
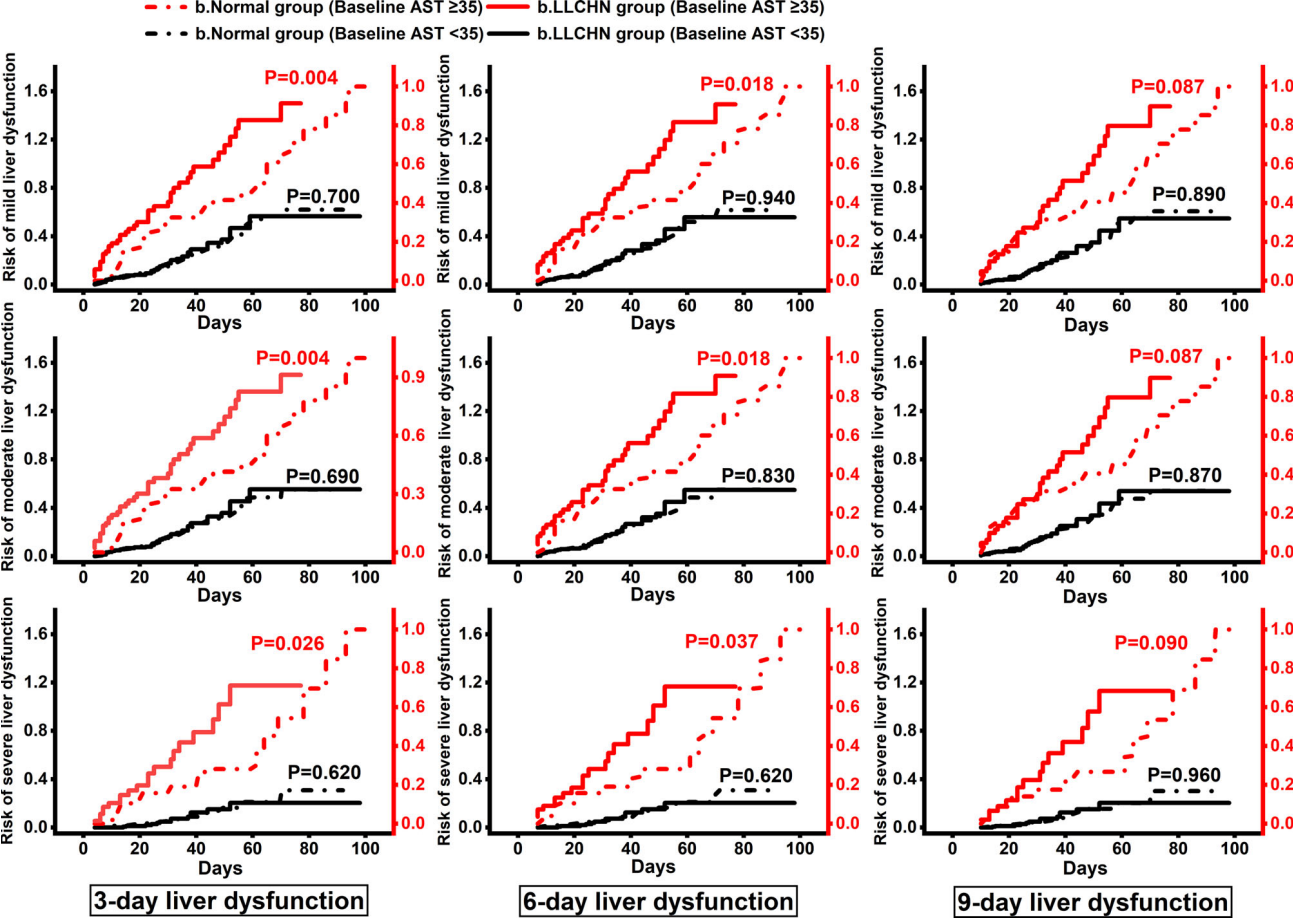
Kaplan–Meier Curves for the cumulative risk of 3-, 6-, and 9-day liver dysfunction in the b.LLCHN group and the b.normal group. Kaplan–Meier curves were divided into two parts by the baseline levels of AST. Participants with the peak values of lymphocyte ratio ≤26.1 and neutrophil ratio ≥62.0 within 3 days of admission were classified as b.LLCHN group, and others were classified as b.Normal group. AST, aspartate transaminase; CI, confidence interval.

The corresponding Kaplan–Meier curves are presented in **Figure 3**. The results of 3- and 6-day liver dysfunction were all significantly different (*p* < 0.05 for all) for the comparison between the b.normal group and the b.LLCHN group in patients with preexisting AST ≥ 35 U/L, but not patients with baseline AST < 35 U/L (*p >*0.6 for all). The results of the 9-day liver dysfunction were not significantly different between the two groups in patients with baseline AST ≥ 35 U/L, but the trends are similar with another two time-points. Similarly, Kaplan–Meier analysis results from patients with baseline AST < 35 U/L also presented no differences between the b.normal group and the b.LLCHN group.

## Discussion

To our knowledge, this is by far the first study to demonstrate the non-linear associations of LR and NR with liver function in COVID-19 patients exposed to antiviral drugs. The lateral dynamic relationships of LR and NR with AST and AST-linked liver dysfunction were U-shaped, with threshold and saturation effects of LR ≤ 26.1 and NR ≥ 62.0, which positively related to the occurrence of liver dysfunction during the observation period. In addition, we observed that baseline LR ≤ 26.1 and NR ≥ 62.0 have a joint effect and a strong positive correlation with 3-, 6-, and 9-day liver dysfunction in COVID-19 patients with preexisting AST ≥ 35 U/L, but not in patients with normal AST levels on admission. COX regression analyses demonstrated that baseline LR ≤ 26.1 combined with NR ≥ 62.0 is an important and independent predictor for the subsequent progression of liver dysfunction in patients with AST ≥ 35 U/L of admission.

In this study, the COVID-19 patients who were exposed to at least one of the following antivirus drugs—LPV, ABI, IFN, or RBV—were selected as the target population due to a combination of reasons: these drugs were recommended as first-line drugs, and most commonly used in China (13, 14, 19); liver damage may be associated with these medications whose hepatotoxicity has been reported by previous studies in different populations (2, 13); and there were few studies on the dynamic changes of liver function in COVID-19 patients exposed to designated antivirus drugs and their hepatotoxicity during the treatment of SARS-CoV-2 infection needs to be considered urgently (20, 21). Our study provided data on serial liver biochemistries, inflammation factors, and antiviral drug usage of COVID-19 patients during hospitalization and the post-discharge isolation period. The data of **Table S3** and **Table 1** show no differences in the antiviral drug usage, but show significant differences in inflammatory factors between the b.normal group and the b.LLCHN group, which illustrated that liver dysfunction in the population of our study, however, may be mainly caused by inflammation, not by antiviral drug exposure.

At present, the pathophysiological foundation of liver damage in COVID-19 patients remains unclear (1). Previous studies reported that inflammatory response affects the incidence of COVID-19-associated liver damage (22–24). However, uncertainty remains regarding whether the proactive identification and management of pre-inflammation are warranted in liver dysfunction prevention in patients with COVID-19. The present study took full advantage of each-point data to examine the lateral relations of LR and NR with liver dysfunction among 1,409 measurements of AST, LR, and NR in 245 patients. The data showed a U-shaped curve from lateral analyses, and the threshold levels of LR and NR on liver function were found. The remarkable differences in AST change among subgroups as defined by LR ≤ 26.1 and NR ≥ 62.0 were observed. Low LR and high NR are related to liver dysfunction events during the lateral observation period. These findings may provide a clue about the underlying pathophysiology of the impact of COVID-19 on the liver.

Liver dysfunction, the risk factor of mortality, is often evident in patients with COVID-19 (4, 25). Of these patients in the current study, 107 (43.7%) experienced mild liver dysfunction, 99 (40.4%) experienced moderate liver dysfunction, and 50 (20.4%) experienced severe liver dysfunction over a median follow-up of 1.9 (IQR, 0.9–4.0) weeks (data not shown). These rates are higher than reported cohorts in China, but lower than those in the USA (12, 21). This inconsistency may result from the higher upper limit of normal for AST compared with ours in the Chinese cohort, and the racial difference with Americans.

In this present study, AST was selected as the dynamic reference index for saturation and threshold effect analysis in our study because the elevation of AST is common and appears to reflect true hepatic injury in COVID-19 patients (12). As expected, the lymphocytes and neutrophils, which are closely related to liver injury in COVID-19 individuals (4), both presented stable U-shaped associations with AST when they were transformed as percentiles. Likewise, the incidences of mild liver dysfunction, moderate liver dysfunction, and severe liver dysfunction were associated with LLCHN in the lateral analyses. Based on these findings, we selected the peak values of LR and NR within 3 days of admission as the baseline to detect the longitudinal associations of the two inflammatory factors with subsequent adverse liver events. Noticeably, among the patients with baseline peak values of LR ≤ 26.1, NR ≥ 62.0, and AST ≥ 35 U/L, the fully adjusted risks of 3-, 6-, and 9-day mild liver dysfunction independently increased at least 5-fold compared to others. Similar trends were observed for moderate liver dysfunction. The 3-, 6-, and 9-day estimates for severe liver dysfunction were not significant in the adjusted II model but significant in the adjusted I model. The insignificant results may be due to the fewer events of severe liver dysfunction after 9 days of admission in this population, limiting our ability to make accurate comparisons. Thus, additional longitudinal large sample size studies with prolonged follow-up, assessing the effects of baseline LR and NR on adverse liver outcomes and considering the modification of time, are required to address this issue.

The dynamic changes of liver function over lymphocytes and neutrophils on COVID-19 individuals have never been reported, as far as we know. As yet, only one study carried out by Lei et al. synchronously assessed the correlations of the normal lower limit of lymphocytes <1.1 × 10^9^/l (OR: 2.21; 95% CI: 1.89, 2.58) and the normal upper limit of neutrophils >6.3 × 10^9^/l (OR: 1.60; 95% CI: 1.31, 1.95) with the incidence of elevated AST but did not assess the lateral dynamic associations of lymphocytes and neutrophils with AST in detail, highly restricting its applicability (4). Furthermore, the study reported by Huang et al. suggested that decreased lymphocytes were independently associated with liver injury (ALT >3×ULN) (21); the trend in this report is similar to ours. However, the definition of decreased lymphocytes in this study was ambiguous. The results of our analyses derived from clear cutoffs of LR and NR, and an appropriate adjustment of 22 covariates, which all related to liver dysfunction (*p <*0.01) or presented a change in regression coefficients of more than 10% (15), avoiding the unmeasured confounding to some extent and making the conclusion of our findings more accurate.

The Kaplan–Meier curves in **Figure 3** also showed that baseline LR ≤ 26.1 and NR ≥ 62.0 revealed significant predictive values for 3- and 6-day liver dysfunction in patients with AST ≥ 35 U/L (*p* < 0.05 for all), but not in patients with normal levels of AST (*p* > 0.6 for all). The significant relationships between LR, NR, and liver dysfunction in COVID-19 patients with abnormal AST proved that the proactive identification and management of pre-inflammation are warranted for liver dysfunction deterioration, especially in COVID-19 patients with preexisting AST ≥ 35 U/L. The mechanism may result from the violent immune response that causes the generation of plentiful inflammatory cytokines, leading to systemic inflammatory response syndrome and further liver ischemia and hypoxia. The low level of Hb in COVID-19 patients with LR ≤ 26.1 and NR ≥ 62.0 (**Table 1**), which may result from its own consumption under the condition of inflammation oxidative stress (26), confirmed this explanation from another perspective. Given the high incidence of liver dysfunction in this current study, we strongly agree with the recommendation of the American Association for the Study of Liver Diseases (AASLD) that liver biochemical indexes should be monitored closely in COVID-19 patients (22).

Our findings provided important clinical and research illuminations. Ours is the first comparatively welled adjustment for potential confounding to show the non-linear associations and threshold effects of LR and NR with liver dysfunction (4). Nowadays, liver dysfunction in COVID-19 patients is prevalent. To investigate inflammation management and monitoring approaches would particularly provide a relevant benefit either for preventing or for salvaging hepatic dysfunction in such settings; a study like ours underlines an urgent need for this topic. Additionally, further studies should focus on prediction models combined with LR ≤ 26.1 and NR ≥ 62.0, and other factors, such as age and BMI, which can efficiently help clinicians identify COVID-19-related liver dysfunction, may alter and improve the therapeutic process program.

Other than the limitations mentioned above, the inherent shortcomings of the retrospective observational single-center study, small sample size, and short-term follow-up make it difficult to address the causality between LR, NR, and liver dysfunction and reach a firm conclusion. However, a comparatively welled adjustment for potential confounding in every multivariate model is thus a trade-off to minimize these biases and confounding. In addition, it would have been interesting to analyze the association between LR, NR, and liver function in our study using an AST ≥ 3×ULN. However, few patients in our clinic-based study had this outcome. Because we used a cut point of AST ≥ 35 U/L for all the adverse liver outcomes, our study demonstrated a severe liver dysfunction and not a liver injury, as defined by the previous report (2).

In conclusion, the U-shaped relationships between LR, NR, and AST and the incidence of AST-linked liver dysfunction were observed in COVID-19 patients exposed to antiviral drugs, with threshold and saturation effects observed in the non-linear relationships. Our data suggest that LR ≤ 26.1 and NR ≥ 62.0 of admission were tightly related to subsequent liver dysfunction and the deterioration of liver abnormalities, especially in patients with preexisting AST ≥ 35 U/L. To improve liver function in COVID-19 patients with abnormal AST, future management strategies should pay more attention to baseline LR ≤ 26.1 and NR ≥ 62.0 cases. Besides, the U-shaped non-linear associations of LR, NR, and liver function may also open new avenues of diagnostic and treatment options, so as to delay the progression of liver dysfunction among COVID-19 patients.

## Data Availability Statement

The original contributions presented in the study are included in the article/**Supplementary Material**. Further inquiries can be directed to the corresponding authors.

## Ethics Statement

This study was approved by the Institutional Review Board of Xixi Hospital. Informed consent was not required due to the retrospective nature of this study.

## Author Contributions

FL and J-FB conceived, designed, and organized the study, interpreted the results, and drafted the manuscript. J-SH helped supervise the research. The other authors contributed to collect and manage the data on-site. All authors contributed to the article and approved the submitted version.

## Funding

This work was supported by the Hepatology (Traditional Chinese and Western Medicine) of Hangzhou Medical Peak Subject and the Hangzhou Science and Technology Development Plan (202004A20).

## Conflict of Interest

The authors declare that the research was conducted in the absence of any commercial or financial relationships that could be construed as a potential conflict of interest.

## Publisher’s Note

All claims expressed in this article are solely those of the authors and do not necessarily represent those of their affiliated organizations, or those of the publisher, the editors and the reviewers. Any product that may be evaluated in this article, or claim that may be made by its manufacturer, is not guaranteed or endorsed by the publisher.
