# Supplementary material for: The Associations of Lymphocyte Ratio and Neutrophil Ratio on Liver Dysfunction in COVID-19 Patients
Source: Front Immunol. 2021 Sep 6;12:717461. doi: 10.3389/fimmu.2021.717461 (PMC8450365; doi:10.3389/fimmu.2021.717461)
Supplement: Supplementary file 1 [file DataSheet_1.docx]

**Supplementary material**

**TABLE S1**|Associations of Covariates with AST, Mild Liver Dysfunction, Moderate Liver Dysfunction, and Severe Liver Dysfunction.

| **Associations of covariates with AST** | | | |
| --- | --- | --- | --- |
| **Covariates** | **exp(*β*)** | **95% CI** | ***P* value** |
| Age **^#^** | -0.02 | (-0.09, 0.05) | 0.632 |
| Female **^#^** | -4.59 | (-6.79, -2.40) | <0.001 |
| BMI **^#^** | 0.57 | (0.27, 0.88) | <0.001 |
| SBP **^#^** | 0.04 | (-0.02, 0.11) | 0.191 |
| Smoking **^#^** | -0.65 | (-3.96, 2.65) | 0.698 |
| Alcohol **^#^** | 4.13 | (1.59, 6.67) | 0.002 |
| Highest temperature **^#^** | 1.83 | (0.41, 3.26) | 0.012 |
| Chest congestion **^#^** | -2.41 | (-6.71, 1.89) | 0.273 |
| Liver cirrhosis **^#^** | 14.79 | (4.50, 25.07) | 0.005 |
| HBV **^#^** | 3.12 | (-1.39, 7.63) | 0.175 |
| **Clinical classification** **^#^** |  |  |  |
| Asymptomatic | Ref. |  |  |
| Mild | 0.98 | (-2.67, 4.63) | 0.599 |
| Moderate | 2.69 | (-0.13, 5.51) | 0.062 |
| Severe | 5.48 | (-0.34, 11.30) | 0.065 |
| WBC (×10^9^/L) **^#^** | 0.70 | (0.11, 1.28) | 0.020 |
| RCP (mg/L) **^#^** | 0.04 | (-0.03, 0.10) | 0.240 |
| SAA (mg/L) **^#^** | 0.05 | (0.01, 0.08) | 0.007 |
| ALP (U/L) **^#^** | 0.06 | (0.03, 0.09) | <0.001 |
| APTT (s) | -0.14 | (-0.75, 0.46) | 0.643 |
| GFR (mL/min) **^#^** | 0.02 | (0.00, 0.04) | 0.051 |
| LppA (mg/L) **^#^** | 0.00 | (-0.01, 0.01) | 0.407 |
| Hb (g/L) **^#^** | 0.17 | (0.11, 0.23) | <0.001 |
| Sodium concentration (mmol/L) | 1.06 | (0.54, 1.59) | <0.001 |
| ABE (mmol/L) **^#^** | 1.05 | (0.31, 1.78) | 0.006 |
| AG (mmol/L) **^#^** | 0.03 | (-0.21, 0.27) | 0.821 |
| **Associations of covariates with mild liver dysfunction** | | | |
| **Covariates** | **OR** | **95% CI** | ***P* value** |
| Age **^#^** | 1.00 | (0.99, 1.01) | 0.992 |
| Female **^#^** | 0.54 | (0.41, 0.71) | <0.001 |
| BMI **^#^** | 1.08 | (1.04, 1.12) | <0.001 |
| SBP | 1.01 | (1.00, 1.02) | 0.015 |
| Smoking **^#^** | 0.81 | (0.53, 1.24) | 0.333 |
| Alcohol **^#^** | 1.26 | (0.94, 1.70) | 0.125 |
| Highest temperature **^#^** | 1.32 | (1.11, 1.56) | 0.001 |
| Chest congestion **^#^** | 0.81 | (0.47, 1.41) | 0.463 |
| Liver cirrhosis **^#^** | 3.22 | (1.22, 8.54) | 0.019 |
| HBV **^#^** | 1.92 | (1.20, 3.06) | 0.006 |
| **Clinical classification** **^#^** |  |  |  |
| Asymptomatic | Ref. |  |  |
| Mild | 1.09 | (0.69, 1.72) | 0.711 |
| Moderate | 1.21 | (0.85, 1.73) | 0.288 |
| Severe | 1.92 | (1.02, 3.64) | 0.044 |
| WBC (×10^9^/L) **^#^** | 1.05 | (0.98, 1.13) | 0.135 |
| RCP (mg/L) **^#^** | 1.01 | (1.00, 1.02) | 0.323 |
| SAA (mg/L) **^#^** | 1.00 | (1.00, 1.01) | 0.223 |
| ALP (U/L) **^#^** | 1.01 | (1.00, 1.01) | <0.001 |
| APTT (s) | 0.99 | (0.92, 1.06) | 0.810 |
| GFR (mL/min) **^#^** | 1.00 | (1.00, 1.01) | 0.004 |
| LppA (mg/L) **^#^** | 1.00 | (1.00, 1.00) | 0.321 |
| Hb (g/L) **^#^** | 1.02 | (1.01, 1.03) | <0.001 |
| Sodium concentration (mmol/L) **^#^** | 1.15 | (1.07, 1.23) | <0.001 |
| ABE (mmol/L) **^#^** | 1.16 | (1.02, 1.32) | 0.024 |
| AG (mmol/L) **^#^** | 1.02 | (0.99, 1.05) | 0.212 |
| **Associations of covariates with moderate liver dysfunction** | | | |
| **Covariates** | **OR** | **95% CI** | ***P* value** |
| Age **^#^** | 1.00 | (1.00, 1.01) | 0.387 |
| Female **^#^** | 0.55 | (0.41, 0.74) | <0.001 |
| BMI **^#^** | 1.13 | (1.09, 1.17) | <0.001 |
| SBP | 1.02 | (1.01, 1.02) | 0.001 |
| Smoking **^#^** | 0.85 | (0.55, 1.32) | 0.474 |
| Alcohol **^#^** | 1.36 | (1.00, 1.85) | 0.047 |
| Highest temperature **^#^** | 1.23 | (1.03, 1.47) | 0.022 |
| Chest congestion **^#^** | 0.92 | (0.53, 1.60) | 0.768 |
| Liver cirrhosis | 2.04 | (0.64, 6.57) | 0.230 |
| HBV **^#^** | 1.83 | (1.11, 2.99) | 0.017 |
| **Clinical classification** **^#^** |  |  |  |
| Asymptomatic | Ref. |  |  |
| Mild | 0.95 | (0.58, 1.56) | 0.844 |
| Moderate | 1.24 | (0.86, 1.79) | 0.257 |
| Severe | 2.16 | (1.14, 4.12) | 0.019 |
| WBC (×10^9^/L) **^#^** | 1.03 | (0.96, 1.11) | 0.357 |
| RCP (mg/L) **^#^** | 1.01 | (0.99, 1.02) | 0.314 |
| SAA (mg/L) **^#^** | 1.00 | (1.00, 1.00) | 0.783 |
| ALP (U/L) **^#^** | 1.01 | (1.00, 1.01) | 0.001 |
| APTT (s) | 0.96 | (0.89, 1.04) | 0.326 |
| GFR (mL/min) **^#^** | 1.00 | (1.00, 1.00) | 0.681 |
| LppA (mg/L) **^#^** | 1.00 | (1.00, 1.00) | 0.438 |
| Hb (g/L) **^#^** | 1.02 | (1.01, 1.03) | <0.001 |
| Sodium concentration (mmol/L) **^#^** | 1.19 | (1.11, 1.28) | <0.001 |
| ABE (mmol/L) | 1.18 | (1.03, 1.35) | 0.019 |
| AG (mmol/L) | 0.84 | (0.74, 0.96) | 0.013 |
| **Associations of covariates with severe liver dysfunction** | | | |
| **Covariates** | **OR** | **95% CI** | ***P* value** |
| Age **^#^** | 1.00 | (0.99, 1.01) | 0.910 |
| Female **^#^** | 0.44 | (0.27, 0.71) | 0.001 |
| BMI **^#^** | 1.21 | (1.14, 1.28) | <0.001 |
| SBP **^#^** | 1.02 | (1.01, 1.04) | 0.001 |
| Smoking **^#^** | 1.44 | (0.82, 2.53) | 0.207 |
| Alcohol **^#^** | 1.30 | (0.81, 2.08) | 0.280 |
| Highest temperature **^#^** | 1.47 | (1.13, 1.91) | 0.004 |
| Chest congestion **^#^** | 1.24 | (0.58, 2.65) | 0.573 |
| Liver cirrhosis **^#^** | 5.54 | (1.70, 18.01) | 0.004 |
| HBV **^#^** | 1.66 | (0.77, 3.58) | 0.193 |
| **Clinical classification** **^#^** |  |  |  |
| Asymptomatic | Ref. |  |  |
| Mild | 0.51 | (0.21, 1.26) | 0.147 |
| Moderate | 1.19 | (0.68, 2.08) | 0.536 |
| Severe | 2.83 | (1.19, 6.72) | 0.019 |
| WBC (×10^9^/L) **^#^** | 1.12 | (1.01, 1.23) | 0.033 |
| RCP (mg/L) **^#^** | 0.97 | (0.93, 1.00) | 0.055 |
| SAA (mg/L) **^#^** | 0.99 | (0.99, 1.00) | 0.139 |
| ALP (U/L) **^#^** | 1.01 | (1.00, 1.01) | 0.004 |
| APTT (s) **^#^** | 0.87 | (0.75, 1.01) | 0.066 |
| GFR (mL/min) **^#^** | 1.00 | (1.00, 1.01) | 0.676 |
| LppA (mg/L) **^#^** | 1.00 | (1.00, 1.00) | 0.797 |
| Hb (g/L) **^#^** | 1.03 | (1.01, 1.04) | <0.001 |
| Sodium concentration (mmol/L) **^#^** | 1.40 | (1.24, 1.57) | <0.001 |
| ABE (mmol/L) **^#^** | 1.46 | (1.16, 1.83) | 0.001 |
| AG (mmol/L) **^#^** | 0.89 | (0.71, 1.11) | 0.301 |

Selection criteria: the covariates selection was based on their regression correlations with liver dysfunction events (P <0.01) or a change in regression coefficients of more than 10%, which was labeled pound (**^#^**).

Abbreviations: exp(*β*), the regression coefficient of continuous covariable on AST; OR, the odds ratio of categorical covariable on liver dysfunction events; AST, aspartate aminotransferase; BMI, body mass index; HBV, Hepatitis B Virus; WBC, white blood cell count; RCP, rapid C-reactive protein; SAA, serum amyloid A protein; ALP, alkaline phosphatase; APTT, activated partial thromboplastin time; GFR, glomerular filtration rate; LppA, lipoprotein A; Hb, hemoglobin; ABE, actual base excess; AG, anion gap.

**TABLE S2**|Threshold Effect of Lymphocyte Ratio and Neutrophil Ratio on AST Using Piecewise-linear Regression Based on Generalized Additive Mixed Model.

|  | | **Exp(*β*) (95% CI)**  **(n=1409)** |
| --- | --- | --- |
| **AST change over LR** | | |
| **One-linear model** |  | -0.17 (-0.31, -0.02) ^*^ |
| **Piecewise-linear model** | LR ≤26.1 | -0.53 (-0.84, -0.22) ^*^ |
|  | LR >26.1 | 0.04 (-0.17, 0.26) |
| **Comparison of models** | Log-likelihood ratio test ^*^ P<0.05 | |
| **AST change over NR** | | |
| **One-linear model** |  | 0.11 (-0.01, 0.24) |
| **Piecewise-linear model** | NR <62.0 | -0.10 (-0.31, 0.11) |
|  | NR ≥62.0 | 0.34 (0.12, 0.57) ^*^ |
| **Comparison of models** | Log-likelihood ratio test ^*^ P<0.05 | |

Exp(*β*) represents the slope of AST (U/L) over LR or NR in segmented groups, obtained with the adjustment of fixed covariates including age, sex, body mass index (BMI), SBP, smoking, alcohol, highest temperature, chest congestion, liver cirrhosis, HBV, and clinical classification on admission, and time-varied covariates including white blood cell count (WBC), rapid C-reactive protein (RCP), serum amyloid A protein (SAA), alkaline phosphatase (ALP), activated partial thromboplastin time (APTT), glomerular filtration rate (GFR), lipoprotein A (LppA), hemoglobin (Hb), sodium concentration, actual base excess (ABE), and anion gap (AG) during the lateral observation period.

Log-likelihood ratio test was used to the comparison of the one-linear model with the piecewise-linear model, below 0.05 indicates piecewise-linear model was a better fit to the data than the one-linear model that assumed a single slope across the entire period of observation.

Abbreviations: LR, lymphocyte ratio; NR, neutrophil ratio.

A result was considered statistically significant when the two-tailed P value was below 0.05, which was labeled asterisk (^*^).

**TABLE S3**| Antiviral Drugs Usage of COVID-19 Patients

|  | **Median (IQR) / Number (%)** | |  |
| --- | --- | --- | --- |
| **Characteristics** | **b.Normal group** | **b.LLCHN group** | ***P* value** |
| Time from infection diagnosis to initiation of antiviral therapy | 13.0 (5.0-27.0) | 13.0 (5.0-28.0) | 0.305 |
| Duration of antiviral therapy | 13.0 (5.0-27.0) | 13.0 (5.0-28.0) | 0.305 |
| Duration of monotherapy | 20.0 (7.0-30.0) | 20.0 (12.0-32.0) | 0.162 |
| Duration of bigeminy therapy | 11.0 (5.0-18.0) | 10.0 (5.0-18.0) | 0.933 |
| Duration of triple therapy | 7.0 (3.0-12.0) | 7.0 (3.0-12.0) | 0.726 |
| Duration of quadruple therapy | 11.0 (10.0-18.2) | 10.0 (5.0-16.0) | 0.390 |
| Monotherapy (%) | 75 (51.4%) | 48 (48.5%) | 0.058 |
| Bigeminy therapy (%) | 98 (67.1) | 68 (68.7%) | 0.176 |
| Triple therapy (%) | 91 (62.3%) | 75 (75.7%) | 0.067 |
| Quadruple therapy (%) | 12 (8.2%) | 8 (8.1%) | 0.669 |

Participants with the peak values of lymphocyte ratio ≤26.1 and neutrophil ratio ≥62.0 within 3 days of admission were classified as b.LLCHN group, and others were classified as b.Normal group.

Abbreviations: IQR, interquartile range.

**TABLE S4**| Characteristics of Fixed Variables and Time-varied Laboratory Variables during the Lateral Observation Period.

| **Fixed variables on admission** | |
| --- | --- |
| Age | 36.0 (27.0-49.0) |
| Female | 95 (38.8%) |
| BMI | 22.8 (20.3-25.6) |
| SBP | 130.0 (117.0-139.0) |
| Smoking | 38 (15.5%) |
| Alcohol | 65 (26.5%) |
| Highest temperature | 37.2 (36.7-37.7) |
| Chest congestion | 11 (4.5%) |
| Liver cirrhosis | 1 (0.4%) |
| HBV | 12 (4.9%) |
| **Clinical classification** |  |
| Asymptomatic | 78 (31.8%) |
| Mild | 36 (14.7%) |
| Moderate | 122 (49.8%) |
| Severe | 9 (3.7%) |
| **Time-varied variables** **during the lateral observation period (No. M=1409)** | |
| **Inflammatory indicators** | |
| WBC (×10^9^/L) | 6.0 (4.8-7.3) |
| RCP (mg/L) | 5.0 (1.0-11.0) |
| SAA (mg/L) ^*^ | 32.0 (9.0-97.2) |
| Lymphocyte ratio (%) | 27.1 (20.7-33.5) |
| Neutrophil ratio (%) | 63.3 (56.0-70.5) |
| **Liver biochemical indicators** | |
| ALT (U/L) | 23.0 (14.0-43.0) |
| AST (U/L) | 23.0 (18.0-30.0) |
| GGT (U/L) | 28.0 (19.0-46.0) |
| TBIL (µmol/L) | 10.8 (8.0-15.2) |
| ALP (U/L) | 77.0 (64.0-94.0) |
| APTT (s) | 28.7 (27.0-30.8) |
| **Others** | |
| GFR (mL/min) | 115.0 (101.0-131.0) |
| LppA (mg/L) | 163.7 (76.3-331.3) |
| Hb (g/L) | 136.0 (123.0-150.0) |
| Sodium concentration (mmol/L) | 138.5 (137.1-139.8) |
| ABE (mmol/L) | 1.0 (-0.2-2.1) |
| AG (mmol/L) | 9.5 (8.3-11.0) |

Data are n (%) or median (IQR) unless otherwise indicated.

Abbreviations: BMI, body mass index; HBV, Hepatitis B Virus; WBC, white blood cell count; RCP, rapid C-reactive protein; SAA, serum amyloid A protein; ALT, alanine aminotransferase; AST, aspartate aminotransferase; GGT, γ-glutamyl transferase; TBIL, total bilirubin; ALP, alkaline phosphatase; APTT, activated partial thromboplastin time; GFR, glomerular filtration rate; LppA, lipoprotein A; Hb, hemoglobin; ABE, actual base excess; AG, anion gap; No. M, the number of measurements.

The asterisk (^*^) represents the level that was above the normal upper limit
